# Effects of Different Concentrations of Arsine on the Synthesis and Final Properties of Polypropylene

**DOI:** 10.3390/polym14153123

**Published:** 2022-07-31

**Authors:** Joaquín Hernández-Fernández, Yoleima Guerra, Esneyder Puello-Polo, Edgar Marquez

**Affiliations:** 1 Chemistry Program, Department of Natural and Exact Sciences, San Pablo Campus, University of Cartagena, Cartagena 130015, Colombia; 2 Centro de Investigación en Ciencias e Ingeniería, CECOPAT&A, Cartagena 131001, Colombia; yoleima.guerra@cecopat.com; 3 Grupo de Investigación en Oxi/Hidrotratamiento Catalítico Y Nuevos Materiales, Programa de Química-Ciencias Básicas, Universidad del Atlántico, Puerto Colombia 081001, Colombia; esneyderpuello@mail.uniatlantico.edu.co; 4 Grupo de Investigaciones en Química Y Biología, Departamento de Química Y Biología, Facultad de Ciencias Básicas, Universidad del Norte, Carrera 51B, Km 5, Vía Puerto Colombia, Barranquilla 081007, Colombia

**Keywords:** arsine, ligands, polypropylene, catalyst, degradation

## Abstract

This article studies the effects of arsine on the synthesis and thermal degradation of 4 samples of virgin polypropylene (PP-virgin) and proposes reaction mechanisms that allow understanding of its behaviour. Different points are monitored during the polypropylene synthesis to perform TGA, DSC, FT-IR, RDX, and MFI analyses later. The content of AsH_3_ in polypropylene varies between 0.05 and 4.73 ppm, and of arsenic in virgin PP residues between 0.001 and 4.32 ppm for PP0 and PP10, increasing in fluidity index from 3.0 to 24.51. The origin of thermo-oxidative degradation is explained by the reaction mechanisms of the Molecule AsH_3_ with the active titanium center of the ZN catalyst and the subsequent oxidation to form radical complexes. OO-AsH-TiCl_4_-MgCl_2_ and (OO-as-OO)_2_ -TiCl_4_-MgCl_2_, which, by radical reactions, give rise to the formation of functional groups aldehyde, ketone, alcohol, carboxylic acid, CO, CO_2_, PP-Polyol, PP-Polyether, and PP-Isopropylethers. These species caused the TG and DTG curves to increase degradation peaks in pp samples.

## 1. Introduction

Arsine (AsH_3_) is a chemical compound of inorganic nature formed by an arsenic atom and three hydrogen atoms, and has a general structure ER_3_ (E = As, N, P, Sb, Bi, and R = H, alkyl, aryl, halogens) and a tetrahedral geometry, with the pair of non-shared electrons located in one of its vertices, which can act as a soft Lewis base [1,2]. In addition, it can bind to transition metals (M), specifically those that have partially filled d-orbitals and with vacant s and p orbitals, and form M-ER_3_ complexes using a covalent bond between the arsine and the metal [1,3]. The coordination of the metals of the d-block of these compounds is given two bonds at once: the donor bond σ and acceptor π. The donor bond σ is established between the pair of nonbonding electrons of the ER_3_ ligand, belonging to the HOMO orbital of the ligand (Highest Occupied Molecular Orbital) and the vacant orbitals of the metal (LUMO, Lowest Unoccupied Molecular Orbital). On the other hand, for the π bond, an action called retro bonding occurs between the filled or half-filled orbitals of the metal (HOMO of the metal) and the anti-bonding σ orbitals of the E-R ligand and/or the empty d orbitals of the heteroatom (E) [4,5]. Arsine complexes for use in catalytic processes are of interest for their organometallic characteristics [6,7,8,9,10], such as their oxidation state, metal coordination number, and the ease by which they can form vacant coordination sites, among others, thereby allowing us to understand its reactivity and behaviour in catalytic systems [11,12]. The use of analysis techniques such as FTIR, X-ray fluorescence, and NMR are necessary for studying these interactions between ligands and metals so that the oxidation of compounds can be evaluated [4,13,14,15].

The effect of AsH_3_ on the synthesis of PP is of importance since, in the polymerization stage of propylene, fundamental raw materials are required, such as Propylene, Ziegler catalyst Natta (ZN), triethylaluminium co-catalyst (TEAL), selectivity control people, hydrogen (H_2_), and nitrogen (N_2_) [16,17], where at least one can act as Lewis acid or have transition metals is its structure. PP synthesis begins with the initiation reaction, which is the activation of the double bond and the active site, followed by a polymerization stage and a polymerization completion stage [18]. The catalyst is titanium (IV) chloride (TiCl_4_), which is a compound with a tendency to form different complexes with a wide diversity of ligands as a result of being a strong Lewis acid [19,20]. In the catalytic system used for the synthesis of PP, the effect of impurities such as this and other components, such as inhibitors of PP polymerization, have been examined and quantified [15,21,22,23,24,25,26,27,28]. In this sense, AsH_3_ is an inhibitor of polymerization reactions. It is observed that its effect is irremediable to the catalytic system and its coordination reaction with the active center of Ti in the catalyst, as well as to the different surfaces of MgCl_2_ and with the alkyl co-catalyst [29,30,31].

In this research, the synthesis of 4 PP is conducted, and specific concentrations of AsH_3_ are dosed at each stage [32] to evaluate its impact on the efficiency of the catalyst. Regarding the PP production rate during the polymerization stage, the effect on the onset of thermal degradation of PP will be evaluated. The significant alterations in the physicochemical properties of PP are of great importance, and have not been observed in previous research. These must be studied so that adequate reaction mechanisms can be explained and proposed in order to help understand the effect of arsine.

## 2. Materials and Methods

### 2.1. Standards and Reagents

For the elaboration of this work, a fourth-generation spherical Ziegler-Natta catalyst with MgCl_2_ support with 3.6% by weight of Ti was used; Diisobutyl phthalate (DIBP) as an in-house donor supplied by Sudchemie, Germany. It was used as a triethylaluminium co-catalyst (98% purity TEAL) from Merck, Germany, diluted in n-heptane. As an external donor, cyclohexyl methyl dimethoxysilane (CMDS) acquired from Merck, Germany, was used. Shazand Petrochemical, Iran, provided polymer-grade propylene. The hydrogen and nitrogen used had a purity of 99.999% [33,34].

We worked with AsH_3_ at 99.999% purity and to guarantee the concentrations of 0.1 and 3.0 ppm in LPG balance, which were required for this research. A dilusor in line was used, which allowed for the creation of the mixtures of AsH_3_ and LPG in the required proportions.

### 2.2. Polymerization Process

**Figure 1:**
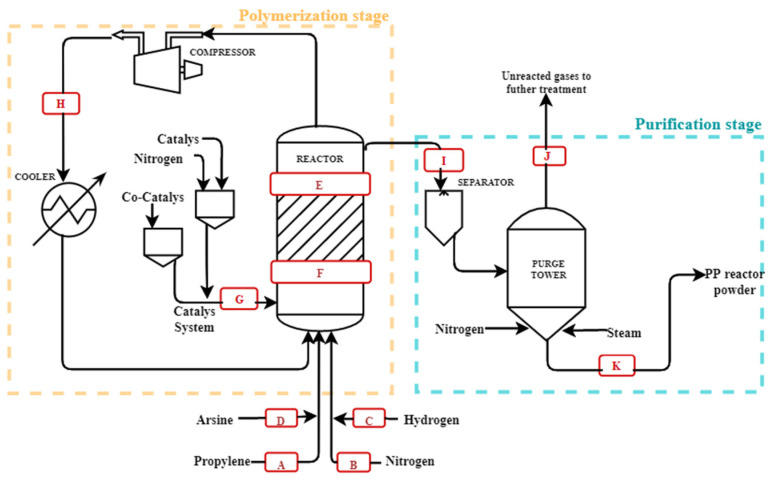
Diagram of the polymerization process and sampling points.

The PP samples were synthesized by polymerization of propylene in the gas phase with the use of a ZN catalyst [35]. The process is shown in Figure 1, and this process consists of a fluidized bed reactor, where nitrogen was initially fed to purge the equipment; subsequently, the propylene and hydrogen that provide the fluidization and absorb the heat of the reaction were fed the catalyst, TEAL, the selectivity control agent, and the nitrogen, in order to carry out the polymerization in discontinuous mode; the quantities are shown in Table 1. This process was conducted at 70 °C and 27 bar pressure. Gases leaving the reactor passed through a compressor to be transported to a heat exchanger where they were cooled and recirculated to the reactor. The resin obtained passed to a purge tower fed with nitrogen and steam to remove traces of hydrocarbons that left the system to obtain a virgin resin [17].

During the process, sampling points were established, as shown in Figure 1. The first points correspond to the feed where samples of propylene were taken (A) that came from liquefied petroleum gases (LPG), nitrogen (B), hydrogen (C), and arsine (D), which came from the same as the propylene from LPG. The following points are those taken from within the reactor; these are composed of gas samples inside the reactor (E), catalyst inside the reactor (F), a sample of the catalytic system (G), and recirculated propylene (H). The last points were taken in the degassing stage, the resin sample that came out of the reactor (I), the gases that were synthesized and removed by virgin PP desorption (J), and the PP obtained from the process (K). Because this process was repeated several times with different concentrations of arsine, a number was assigned to each of them, with zero as the target.

Throughout the process, sampling points were established, as shown in Figure 1. These were found in the feed, in the reaction system, and in the degassing stage. Table 2 describes the sampling points and the identification of the samples; because this process was repeated several times with different concentrations of arsine, a number was assigned to each of them, with zero as the target.

### 2.3. Analysis

#### 2.3.1. Thermogravimetric Analysis-TGA

To determine the effects of arsine on the thermal degradation of the PP samples obtained, the Perkin Elmer TGA7 equipment performed a thermogravimetric analysis (TGA) at a heating rate of 20 °C min^−1^ and under an N_2_ flow rate of 60 mL min^−1^. For this analysis, 10 mg samples were used to obtain the TG and DTG curves [36,37].

#### 2.3.2. Differential Scanning Calorimetry Analysis-DSC

A differential heat scanning (DSC) analysis was performed on a DSC Standard Cell RC. To ensure an identical thermal history, the sample was heated from 0 °C to 230 °C at a rate of 10 °C min^−1^ and subsequently cooled from 230 °C to 0 °C at the same speed.

#### 2.3.3. Melt Flow Index-MFI

The melt flow index (MFI) was measured using a Tinius Olsen MP1200 plastometer. The temperature inside the cylinder of the plastometer was 230 °C and a 2.16 kg piston was used to displace the melt. After the MFI data was obtained, the average molecular weight for each of the PP samples was evaluated using the Bremner approximation [38], which was obtained by correlations that were formulated by the study of commercial polypropylene samples with MI values between 0.7 and 12, measuring the variation in the molecular weight distribution by SEC, using differential refractive index and low-angle laser light scattering detectors [39].

M_W_^3^^.7^ = 1675/((MFI230 °C 2.16 kg) × 10^−21^) (1)

#### 2.3.4. X-ray Fluorescence

This analysis was performed on Malvern’s Axios FAST analytical equipment, which allowed us to see the concentration of arsenic and arsine present at the sample points.

#### 2.3.5. Infrared Fourier Transform Analysis-FT-IR

Fourier transform infrared spectroscopy (FT-IR) was used to determine the most significant structural changes in the PP matrix and the ZN catalyst due to the reaction of ZN Ti with AsH_3,_ and to evaluate how this interaction affected the thermal stability of PP. FT-IR analysis was performed using a Nicolet 6700 (Thermo Scientific) infrared spectrometer using the attenuated total reflectance (ATR) method. The samples were analyzed in the form of films; these films were obtained by compression moulding of the PP in a CARVER 3895 hot press to obtain 300 mm diameter films of ≈100 μm thickness.

#### 2.3.6. Gas Chromatography Analysis with Selective Mass Detector (GC-MS)

**Figure 2:**
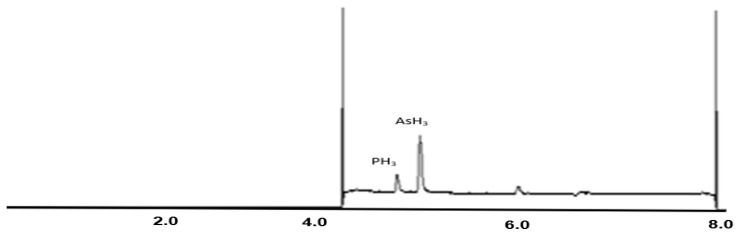
Arsine chromatogram.

A GC-MS 7890B from Agilent technologies was used for arsine analysis [36]. Figure 2 shows the chromatograms of the standards for AsH_3._

#### 2.3.7. Headspace-GC-MS

PP samples were analyzed using an Agilent 7694E Headspace Sampler with the following conditions, GC cycle time: 60 min, furnace: 150 °C, transfer line: 175 °C, loop: 175 °C, vial equilibrium time: 60 min, stirring speed: off, loop size: 1.0 mL, loop filling time: 0.2 min, loop equilibrium time: 0.02 min, injection time: 0.3 min, pressurization time: 0.4.

## 3. Results

### 3.1. Polymerization Process

Polymerization occurs in three stages. The first is the formation of the active site consisting of the alkylation of a pentacoordinate Ti (III) ion for the titanium atom that is on the surface of the layers of the α-TiCl_3_ [40,41] and the activation of the double bond of the monomer. This is given by the coordination of the monomer when establishing a giving bond of electrons p of the double bond with the empty orbital of the transition metal. During the reaction, an intermediate is formed where the unsubstituted carbon binds to the transition metal and the other to the alkyl group [18]. The second stage is the propagation of the monomer, where the active center is linked to the transition metal-alkyl, formed by the constitution of a coordination complex between the growth chain, the added monomer, and the active center of the catalyst [41]. The third stage is the culmination of polymerization, which can be achieved with the separation of hydrogen β, with the removal of a hydride, or by deactivation of the complex using H_2_ [41,42]. The addition of arsine during polymerization can affect this process due to its ability to bind to transition metals such as Ti present in the catalyst, inhibiting its effectiveness. Thus, in this work, samples are taken at strategic points that allow for the measurement of the content of this compound and for the proposition of a reaction mechanism, so that the behavior of the PP-virgin arsine and its effects can be determined.

#### 3.1.1. Quantification of Arsine at the Polymerization Stage

The variation of the concentrations of arsine presented in the samples taken at the different selected points of the polymerization process is shown in Figure 1, where the points detailing the presence of these components can be observed. Point D is the initial concentration of arsine introduced to the process, which varies from 0.05 to 4.73 ppm, referring to the presence of impurities not removed during the purification stage of the refinery grade propylene, which translates to a failure of the petrochemical plants that supply this compound. Point H refers to the recovered propylene; the values obtained are equal to 0, which means that AsH_3_ was adsorbed or absorbed during the polymerization reaction. Point E shows the gaseous propylene found in the reactor; the presence of AsH_3_ at this point indicates that the compound is part of the reaction or interacts in the formation of the polymer being adsorbed, which had been confirmed at point H. Therefore, there must be a reaction mechanism between the catalyst and the arsine that allows the latter to act as a component of the polymerization reaction, which is possible due to the arsine’s ability to form M-ER_3_ bonds, binding to the transition metal of the active center of the catalyst affecting the polymerization reaction, and due to the efficiency of the catalyst.

#### 3.1.2. Reaction Mechanisms of AsH_3_ Proposed during Polymerization: Reaction of Residual AsH_3_ with TiCl_4_/MgCl_2_

**Figure 3:**
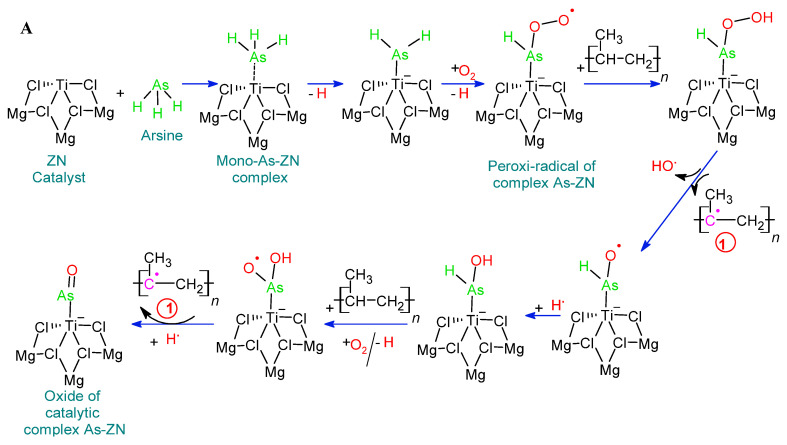

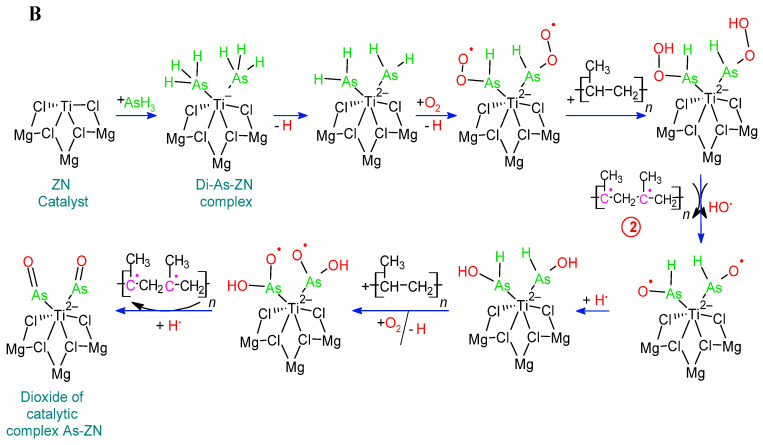
Reaction mechanism of residual AsH_3_ with TiCl_4_/MgCl_2_.

In the mechanism proposed in Figure 3, AsH_3_ competes with the propylene monomer for the active site of Ti. First, a complex π is formed by coordinating the AsH_3_ with the Ti of the TiCl_4_/MgCl_2_ complex. The AsH_3_-Ti interaction is conducted via the interaction of the electropositive Ti with the free electron pair of arsenic, which predominates the electrons of the π bond of propylene. The formation of a complex π in Ti-propylene has no barriers and is accompanied by an energy gain less than that of AsH_3_-Ti, so the latter reaction is predominant. The propylene insertion barrier varies between 6–12 kcal mol^−1^ [43,44,45,46]. The high probability of occurrence of the insertion reaction is supported by thermodynamics, since a favoring of approximately 20 kcal mol^−1^ is observed [30,47,48]. In the PP synthesis process, the polymer chain’s propagation takes place by moving propylene into Ti-PP, where the PP-alkyl chain has the olefin inserted [47,49]. The PP chain’s growth is affected when Ti’s active centre reacts with inhibitors of different polarities. The occurrence of these reactions depends on energetic factors. The coordination of the different inhibitors to the Ti Center on the surface of MgCl_2_ is favored by 27.2 kcal mol^−1^ for Ti-H_2_O, 15.1 kcal mol^−1^ for Ti-H_2_S, 8.4 kcal mol−1 for Ti-CO_2_, 13.1 kcal mol^−1^ for Ti-O_2_, and 30.6 kcal mol^−1^ for Ti-CH_3_OH interactions [26]. Since AsH_3_ exhibits polarities intermediate to the poisons mentioned above, it is expected that its values are within the ranges discussed above. As shown in Figure 3, AsH_3_ interferes with the formation of propylene complexes and their insertion. It is worth mentioning that the interactions of these impurities, including AsH3 with the active centers of Ti, are reversible when these inhibitors are removed from the system, and thereafter, the active center of Ti resumes polymerization [50].

#### 3.1.3. Discussion of FTIR Analysis

**Figure 4:**
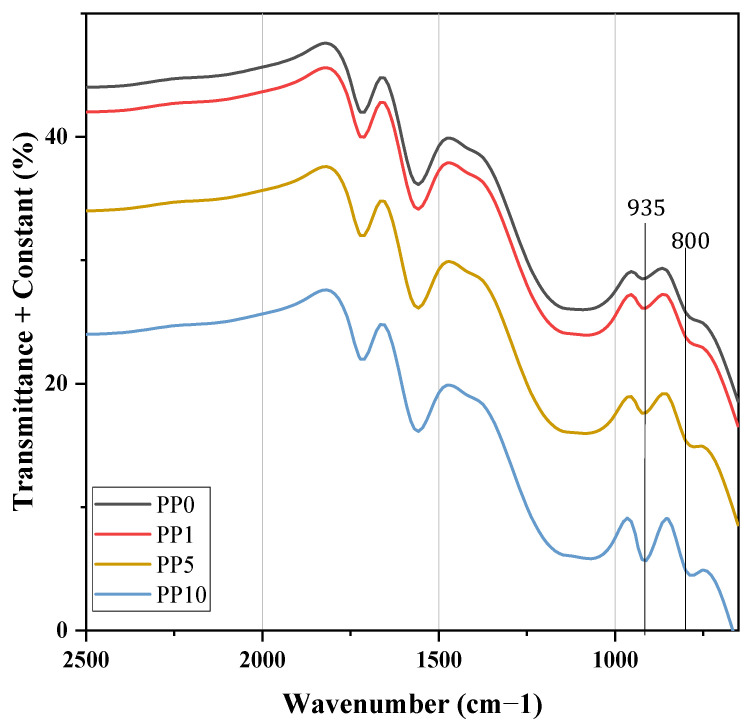
Infrared spectra of the catalyst with distinct levels of arsine.

Figure 4 shows the catalyst’s infrared spectra with various arsine levels used during the synthesis of PP. The above identifies species of oxidized arsenic in the As-ZN complex, thus supporting the mechanisms prepositioned in Figure 3. Concerning the fundamental vibration As-O and As = O in FTIR, the force constants of an As-O bond estimated by the use of Gordy’s rule are 8.7 × 105 and 4.6 × 105 cm^−1^ dinas for a single bond and double bond, respectively [51]. When substituted in the fundamental equation for a harmonic oscillator, these values give VAs-O 964 and 776 cm^−1^ for a “pure” single bond and a double bond, respectively. The values for the fundamental vibration As-0 and As = O were, in most cases, within 10–40 cm^−1^ of 920 cm^−1^ the average of the frequencies calculated for a single and double bond [51,52]. The observed frequency suggests a higher bond order than one that may well result from the subsequent donation of the pπ electrons of the oxygen atom using the empty dπ orbitals of the arsenic atom. Figure 4 shows that the band’s growth between 800 and 970 cm^−1^ is associated with the increase in the concentration of oxidized arsenic atoms in the As-ZN complex, and an increase in the area of these peaks is observed, being greater for the K10 sample. This last catalyst sample was the one that was in contact with the highest levels of AsH_3_. The spectra indicate that the oxidized arsenic that is part of a chemical complex within the catalyst was higher when AsH_3_ levels increased.

**Figure 5:**
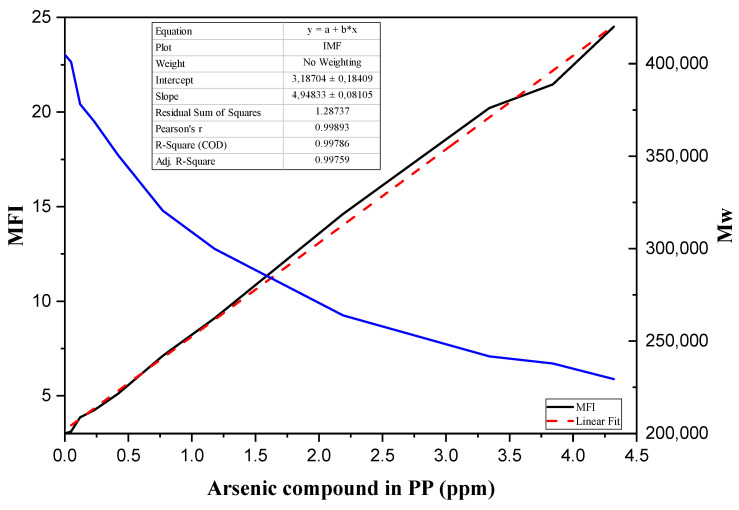
Relationship between the arsenic content in the PP and the MFI, and the variation of the M_w_.

Arsenic of AsH_3_-TiCl_4_-MgCl_2_ is oxidized in the presence of oxygen atoms to form a complex of radicals O-O-AsH_2_-TiCl_4_-MgCl_2_, which are the initiators of the degradation of wasting PP. In Figure 5, the effect of this complex of radicals O-O-AsH-TiCl_4_-MgCl_2_ is shown on the beginning of the degradation of PP, which consists of the abstraction of hydrogen in the PP-virgin, generating a stable tertiary radical, which, when reacting with a molecule O_2_, will produce a Peroxy-PP-virgin radical, which extracts hydrogen from a neighboring virgin-PP chain, thereby forming the virgin-PP-Hydroperoxide. The latter, after a homolytic rupture, will give rise to the Alkoxy-PP-Virgin radical [53,54,55,56]. The radical complex of O-O-ash-tiCl_4_-MgCl_2,_ after the abstraction of hydrogen, forms the peroxy complex H-O-O-AsH-TiCl_4_-MgCl_2_ that, by hemolysis, forms the radical O-AsH-TiCl_4_-MgCl_2_. The latter can perform successive abstractions of hydrogen from the tertiary carbon of the PP-virgin chain to continue increasing its degradation or reaction with hydrogen radicals to end the radicalization process and form the stable complex HO-AsH-TiCl_4_-MgCl_2_. This last complex also participates in the radical reaction with PP-virgin and with oxygen atoms to form the radical tertiary polymer and the intermediate complex HO-As-OH-TiCl_4_-MgCl_2_ that, by dehydration, forms the new adduct O = As-TiCl_4_-MgCl_2_. Figure 3b shows the reaction of two molecules of AsH_3_ with the active center of Ti of the Catalyst ZN, to form the complex (AsH_3_)_2_-TiCl_4_-MgCl_2_ that subsequently oxidizes in the presence of oxygen atoms to form the radical complex (O-O-As-O-O-)_2_-TiCl_4_-MgCl_2_, which are the initiators of the degradation of PP-virgin residues by abstraction of multiple tertiary carbon protons, by the formation of multiple tertiary radicals of the PP-virgin chain and the intermediate complex HO-As-OH-TiCl_4_-MgCl_2_ that, by dehydration, forms the new adduct O = As-TiCl_4_-MgCl_2_.

### 3.2. Effects of Arsine on the Properties of PP

#### 3.2.1. Effects of Arsenic Content on Virgin PP MFI

The effects of the arsenic content in the samples are reflected in the change of the MFI obtained for each of them, presenting a linear correlation with an R^2^ of 0.99786; where the concentration of income of AsH_3_ increases, so does the MFI, as shown in Figure 5. This means that the arsine from impurities (point A) participates in the polymerization reaction, forming a stable complex present in the polymer chain that changes some properties of the resin obtained, including the M_w_ of the samples, which was calculated using the formula proposed by Brenner. Its variation is shown in Figure 5, demonstrating that as the arsenic content present in the resins increases, the MFI decreases.

Consequently, small chain scissors and fractures of the polymer structure during degradation are very frequent, occurring as the average molecular weight decreases and the MFI increases. It should be noted that the residue contents of the catalyst, such as Ti, Al, Cl, and Fe, do not present significant differences, thus the changes presented in this paragraph are not generated by residues other than arsine, as has been presented in other studies [36,57].

From these data, it can be inferred that, during the reaction, the arsine reacts with the ZN catalyst. During the development of this work, a reaction mechanism was proposed where it was evident that the arsine competed with the propylene molecule in the polymerization stage, reacting with the active center of the ZN that influences the growth of the polymer chain and presenting evidence of the high reactivity of the catalyst.

#### 3.2.2. Degradation of Residual PP

**Figure 6:**
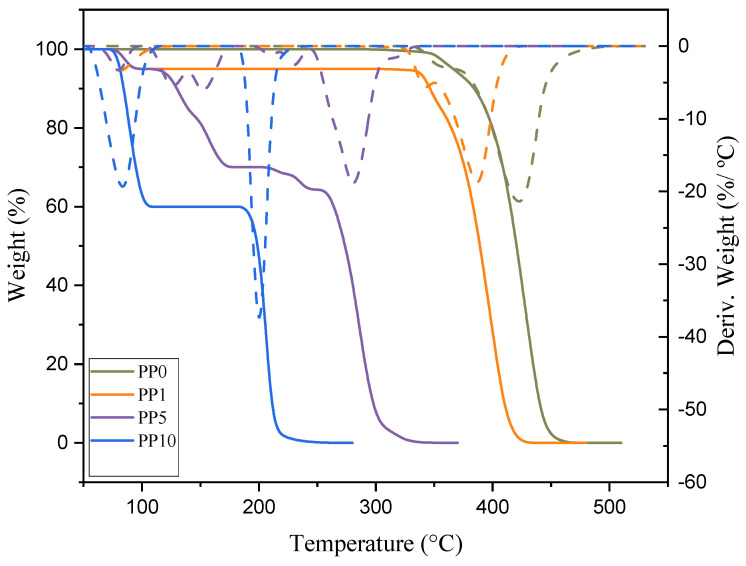
TGA and DTG of PP samples.

The thermal degradation of the samples was evaluated by TGA and DSC, the data obtained show a decrease of the thermal stability of the material as the arsenic content increases. These results are presented in Figure 6. The thermal behavior of the K0 sample does not have a significant variation, as presented in the literature [19]. K0 and K1, which have a zero and very low content of arsenic, respectively, in their matrix, have a peak rupture at 390 and 430 °C with a mass loss of 22%.

**Figure 7:**
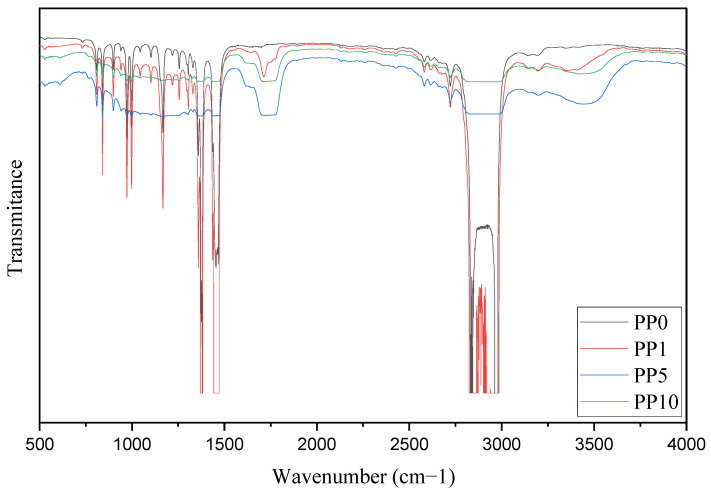
Carbonyl index of PP samples.

The addition of AsH_3_ in the polymerization implies marked changes in the performance and macromolecular structure of the final material obtained, demonstrating that their molecular weight and thermal stability are too low to have functional groups different from the hydrocarbon. Previous research has demonstrated a link between the polymer’s low molecular weight and photooxidative degradation, as indicated by the carbonyl index in the 1712 cm^−1^ band of FTIR (Figure 7), which is impacted when the concentration of arsine and arsenic in the sample increases [57,58,59]. To understand this behavior, a reaction mechanism is proposed to explain the reduction in the physicochemical properties of PP when it has arsine content.

### 3.3. Degassing Process

The degassing process is the second stage of the process, in which the resin passes when leaving the reactor. This consists of the purge tower, where nitrogen and value are fed to remove the hydrocarbons, and the adsorption or absorption of arsine by the resin obtained is confirmed [35].

#### 3.3.1. Identification and Quantification of Arsine in the Degassing Stage of PP Resin

Table 3 shows the concentrations obtained from the different compounds during the degassing stage taken at the available sampling points together with the input data for the overall process. Point I: the samples obtained from this point are of newly polymerized resin, which contains arsine either absorbed or adsorbed. Point J: volatile compounds are removed, having values equal to 0 for the arsine content, which allows us to rule out the absorption of both arsine and arsenic at the interface of the polymer. At this point, the other compounds formed during polymerization that are purged are also determined, which allows us to determine the effect of the arsine on their composition. Finally, point K: showing that the resin obtained has the content of the two compounds, therefore, the products obtained from these processes are considered waste and may present variations concerning virgin resins that can contribute to the degradation of the material, thermal stability, and fluidity index, among others. The study of the effects on these properties allows us to determine applications for these generated residues.

The compounds determined during this stage present a significant increase concerning the target; this increase may be related to the increase in the content of arsine fed in the polymerization stage. If we take point J as a reference, where most of these gaseous compounds are removed, it can be seen that the values are 3.5 to 5.27 times higher for the sample J1 concerning the white (J0); in the case of the J10 sample, the values are between 9.29 and 20.4 times higher with respect to the white, being alcohol the compound, where the greatest increase is shown. In addition, as the arsine content in the feed increases, the formation of CO production is promoted.

#### 3.3.2. Mechanisms of Reaction of Peroxy Radicals in Arsenic Complexes to Form the VOCs Identified in PP

**Figure 8:**
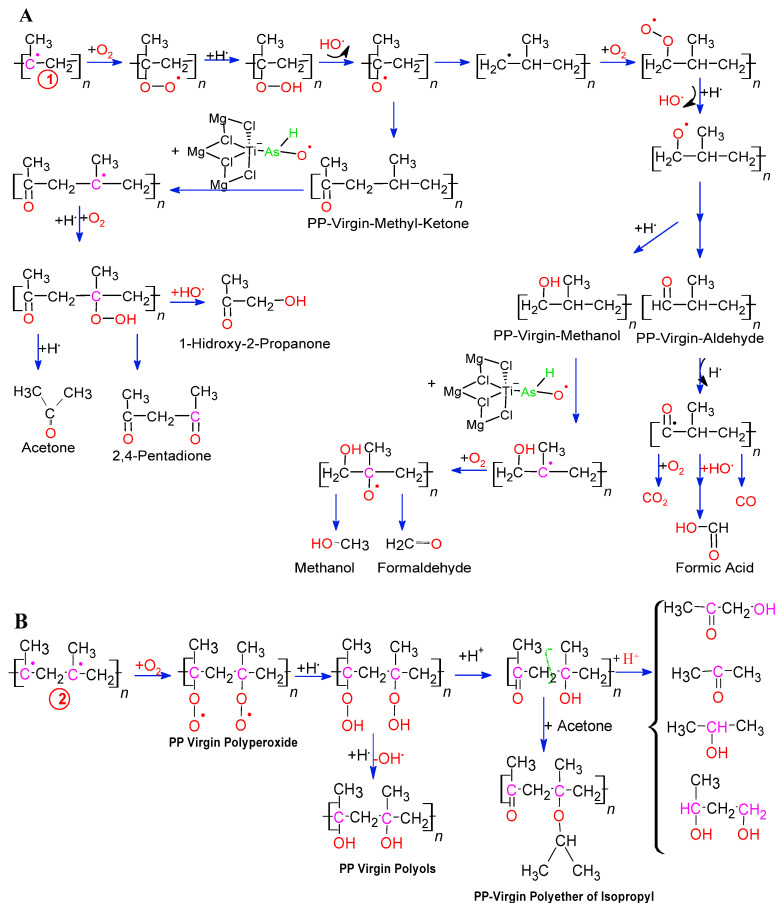
Mechanisms of reaction of peroxy radicals in arsenic complexes to form VOCs.

The Alkoxy-PP-virgin radical that has formed undergoes a homolytic rupture of the simple bond adjacent to the carbon of the oxygenated radical. This homolysis forms the methyl-PP-virgin ketone and the radical PP, as seen in Figure 8. The radical O-AsH-TiCl_4_-MgCl_2_ attacks the tertiary carbon of the methyl-PP-virgin ketone, to abstract the hydrogen atom and thus propagate the oxidation of the more stable carbon [58]. This new radical reacts with the oxygen atom to form the β-Hydroxyperoxy Methyl-PP-virgin Ketone, 2,4-pentanedione, 1-Hydroxy-2-propanone, and acetone. The latter two are formed after homolytic cleavage of another tertiary carbon bond, the formation of the acetone radical, and subsequent reactions with the hydroxyl and hydrogen radicals. On the other hand, the PP radical at the end of the chain reacts with O_2_, giving rise to PP-hydroperoxide, to the PP-Alkoxy radical, and then to methanol-PP-virgin, which, by abstraction of the tertiary carbon proton by the radical O-AsH-TiCl_4_-MgCl_2,_ forms an intermediate radical that, when reacting with O_2_, decomposes into methanol and formaldehyde. Methanol-PP-virgin gives rise to Aldehyde-PP-virgin that, by homolysis of the H-C bond of the terminal carbonyl group, forms the Carbonyl-PP-virgin radical, which in turn, by reactions with hydroxyl radicals and hydrogen, gives rise to CO, CO_2_ and formic acid, as shown in Figure 8.

The strong relationship between the concentration of AsH_3_ residues and the arsenic content within the PP-virgin matrix with the MFI, TG, and DTG shows that increasing the concentration of AsH_3_ drastically decreases the stability of PP, allowing us to propose an alternative reaction mechanism to that shown in Figure 3; a mechanism that comes even closer to the behavior of K10. Figure 8 shows the reaction of two Molecules AsH_3_ with the active center of Ti of the catalyst ZN, to form the complex (AsH_3_)_2_-TiCl_4_-MgCl_2_ that is subsequently oxidized in the presence of oxygen atoms to form the radical complex (O-O-As-O-O-)_2_-TiCl4-MgCl_2_, which are the initiators of the degradation of PP-virgin waste by abstraction of multiple tertiary carbon protons, by the formation of multiple tertiary radicals of the virgin-PP chain. These will react with the oxygen atoms by forming PP-Virgin poly peroxide that supports increased PP fluidity and low TG and DTG values. The poly peroxide of PP-Virgin, due to its chemical structure, has the appropriate stereochemistry to form hydrogen bonds with another poly peroxide of the PP-Virgin to give rise to a macromolecule of greater polarity, and in an acid medium, creates polyols of PP-virgin. Figure 8 also shows that PP-virgin polyoxide, by the effect of hydrogen and hydroxyl radicals, forms PP-virgin polyols with a carboxyl group at the end of the chain. The latter experiences homolytic ruptures and dehydration. In homolysis, it gives rise to acetone, 1-Hydroxy-2-propanone, isopropyl alcohol, and 1,2-Propanediol, and in dehydration, it forms stable tertiary carbocations, which, when reacting with different alcohols and polyols, gives rise to macromolecules of PP-virgin ether type.

This study showed the impact that certain contaminants may have on the manufacture of polymers such as polypropylene. The selection and analysis of raw materials is critical to preventing this sort of reaction from happening during the industrial synthesis of PP. With this research, we expect that the purity of any raw materials will be thoroughly examined before application.

## 4. Conclusions

After the analysis of the samples obtained, it is possible to define the effect that the arsine has on thermal stability and other properties of PP, in addition to defining how an increase of the content of this chemical compound can further enlarge the difference in thermal stability, molecular weight, and MFI between the samples and standard; the first two decreasing and the last increasing. Likewise, the possible reaction mechanisms that occur between the arsine with the active titanium center of the ZN catalyst and the subsequent oxidation to form radical complexes of O-O-AsH-TiCl4-MgCl_2_ and (O-O-As-O-O-) _2_-TiCl_4_-MgCl_2,_ which, by radical reactions, give formation to aldehydes, ketones, alcohols, carboxylic acids of functional groups, CO, CO_2,_ PP-Polyol, PP-ethers, and PP-Isopropyl ethers, can be defined.

## Figures and Tables

**Table 1:** Reagents for polymerization.

Run	Catalyst [kg/h]	Propylene [TM/h]	TEAl ^1^ [kg/h]	Hydrogen [g/h]	Nitrogen	Arsine [ppm]	Selectivity Control Agent [mol/h]	T [°C]	Pressure [bar]
0	5	1.2	0.25	30	.	0	1	70	27
1	5	1.2	0.25	30	.	0.05	1	70	27
5	5	1.2	0.25	30	.	0.84	1	70	27
10	5	1.2	0.25	30	.	4.73	1	70	27

^1^ triethylaluminium co-catalyst.

**Table 2:** Identification and sampling points.

Stage	Point	Substance	State of Origin	Identification
Feeding	A	Propylene	LPG	A0, A1, A5, A10
	B	Nitrogen	Gas	B0, B1, B5, B10
	C	Hydrogen	Gas	C0, C1, C5, C10
	D	Arsine	LPG	D0, D1, D5, D10
Reaction	E	Gases	Inside the reactor	E0, E1, E5, E10
	F	Catalyst	Inside the reactor	F0, F1, F5, F10
	G	Catalyst	Catalytic system	G0, G1, G5, G10
	H	Propylene	Recovered	H0, H1, H5, H10
Degassing	I	Resin	Exits the reactor	I0, I1, I5, I10
	J	Gases	Retired in the purge	J0, J1, J5, J10
	K	Polypropylene	Dust	K0, K1, K5, K10

**Table 3:** Compounds determined in the degassing stage.

Compound (ppm)	Point I				Point J				Point K			
	I0	I1	I5	I10	J0	J1	J5	J10	K0	K1	K5	K10
AsH_3_	0	0	0	0	0	0	0	0	0	0	0	0
Alcohol	10.1	50.2	81.5	180.1	15.2	80.2	100	310.1	5.1	7.5	20.1	50.5
Ketone	19.5	80.2	160.1	220.1	30.2	130.1	220	280.7	4.5	10.6	31.5	45.5
Aldehydes	13.2	60.1	121.5	242.7	25.2	91.5	172	270.1	7.1	11.7	22.3	21.2
Acid	20.1	97.1	210.1	351.7	32.1	161.2	308	425.1	4.5	23.4	41.5	33.3
CO	0	0	0.2	1.1	0	0	0.4	3.2	0	0	0	0.1
CO_2_	0.1	1.5	5.5	11.3	1.2	2.1	8	20.2	0	0.1	1.2	1.9

## Data Availability

The data presented in this study are available on request from the corresponding author.
